# Fecal microbiota transplantation in chronic liver disease: Current and future state of the art

**DOI:** 10.1097/HC9.0000000000000927

**Published:** 2026-03-20

**Authors:** Patricia Bloom, Sahil Khanna

**Affiliations:** Division of Gastroenterology and Hepatology, Mayo Clinic, Rochester, Minnesota, USA

**Keywords:** Microbiome therapeutic;, living bacterial product;, cirrhosis; dysbiosis

## Abstract

Chronic liver diseases are associated with changes in gut microbiome composition and function. Early data suggest that fecal microbiota transplantation (FMT) may treat several chronic liver diseases, especially cirrhosis, hepatic encephalopathy, and alcohol-associated liver disease. Well-powered and multisite studies are needed to better understand which indications and subpopulations hold promise for FMT. At present, there is variability in the screening, processing, and administration of FMT. Some of this variability is inherent to the nature of FMT, but some of the variability could be standardized to optimize safety and efficacy. Ultimately, we may find that narrowed and donor-independent microbiome therapeutics are superior tools to provide a consistently effective result in chronic liver disease. Regulation of FMT for chronic liver disease indications in the United States will continue to require the rigid regulatory framework of other drugs, requiring an Investigational New Drug (IND) application.

## INTRODUCTION

Chronic liver diseases are associated with alterations in gut microbiome composition and function.^1^,^2^,^3^,^4^ Microbial metabolites reach the liver via the portal vein; thus, gut microbiome changes may directly contribute to the development and progression of liver disease. Fecal microbiota transplantation (FMT) is the transfer of stool from a healthy donor to a recipient, and has emerged as a potential management strategy in several chronic liver disease states. FMT has been shown to improve gut microbiome diversity and function, closer to normal, and thus has the potential to improve the downstream impact on liver diseases.

**FIGURE 1:**
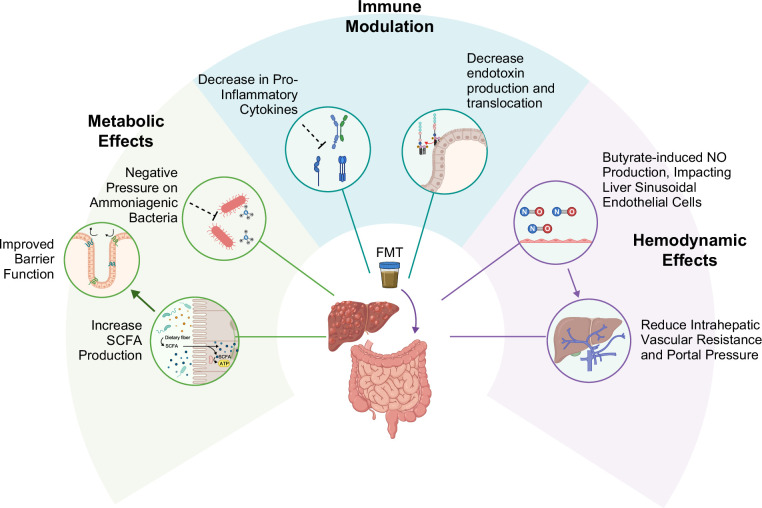
Mechanism of action of FMT in chronic liver disease. FMT in chronic liver disease impacts several physiologic pathways in 3 main categories. First, FMT modulates gut metabolic pathways, especially the production of short-chain fatty acids (SCFAs), which impacts gut barrier integrity. Second, FMT alters immune cell populations and decreases pro-inflammatory cytokines. Finally, FMT-induced butyrate generation may lead to a signaling cascade that results in increased nitric oxide production, altered liver endothelial cell function, and subsequently reduces portal hypertension. Created in BioRender. Bloom, P. (2026), https://BioRender.com/m1veep0. Abbreviations: FMT, fecal microbiota transplantation; NO, nitric oxide.

Several mechanisms have been proposed to explain how FMT may influence liver disease (Figure 1). First, FMT modulates gut metabolic pathways, including the production of ammonia, short-chain fatty acids (SCFAs), phenylalanine, choline, and glycerolipid metabolism in recipients.^5^,^6^,^7^,^8^ These metabolites can affect gut barrier integrity, including SCFAs, which provide an energy source to gut epithelium and increase tight junction protein synthesis and decrease paracellular epithelial permeability.^9^ This improved gut barrier integrity may prevent translocation of ammonia, bacteria, and other bacterial products, which may directly impact hepatic function, renal and muscle physiology, and neurocognitive outcomes, thereby influencing hepatic and extrahepatic manifestations of disease. Second, FMT alters immune cell populations and activity within the gut, liver, and periphery, potentiating immune tolerance.^7^,^10^,^11^ Studies in different liver diseases have demonstrated a decrease in pro-inflammatory cytokines with FMT, including IL-6, IL-17A, TNF-α, TGF-β, and endotoxin.^7^,^11^ Finally, FMT-induced butyrate generation may lead to a signalling cascade that results in increased nitric oxide production, altered liver endothelial cell function, and subsequently reduced portal hypertension.^12^

Despite the promising research conducted to date, substantial gaps remain in our understanding of altering the microbiome in chronic liver disease via FMT or standardized microbiome-based therapies. This review summarizes current evidence regarding its utility, processing, and regulatory considerations, and explores future directions for its clinical application.

## EVIDENCE SUPPORTING USE OF FMT IN CHRONIC LIVER DISEASE

**FIGURE 2:**
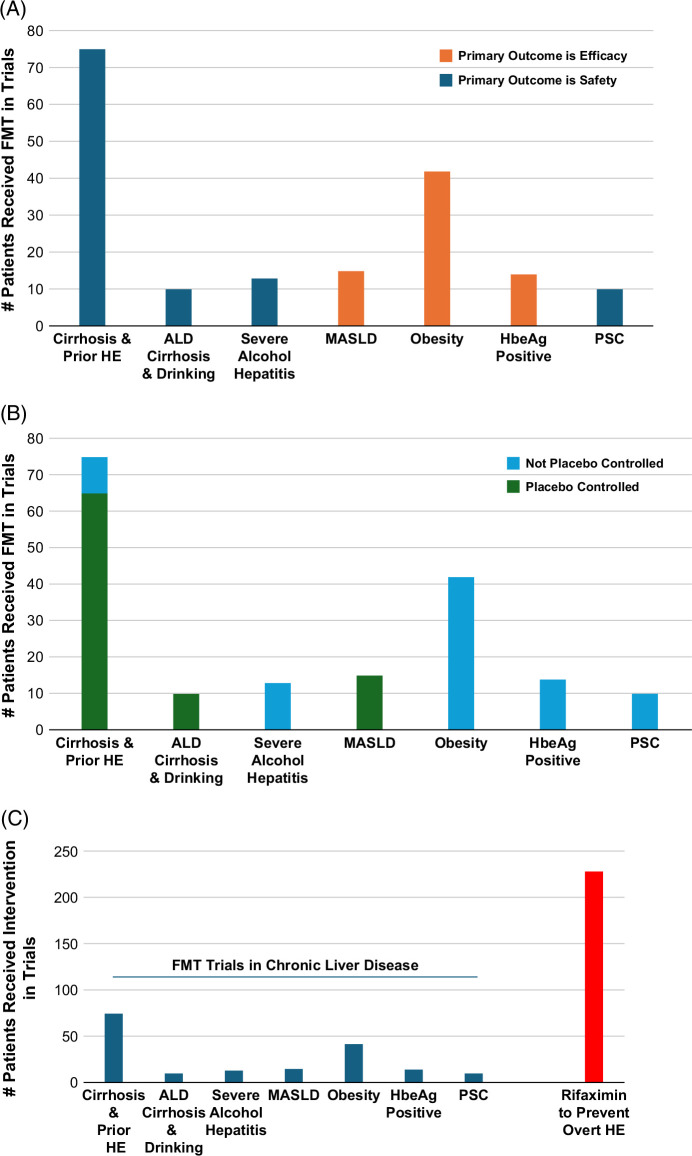
Clinical evidence of FMT across chronic liver diseases. (A) Number of patients who received FMT in trials of chronic liver disease by indication, comparing those with a primary endpoint of safety versus efficacy. (B) Number of patients who received FMT in trials of chronic liver disease by indication, comparing those in a placebo-controlled trial versus those not. (C) Number of patients in chronic liver disease trials, comparing those who received FMT in a trial to those who received rifaximin in trials to prevent recurrent overt HE before rifaximin was approved. Abbreviations: ALD, alcohol-associated liver disease; FMT, fecal microbiota transplantation; HBeAg, hepatitis B e antigen; HE, hepatic encephalopathy; MASLD, metabolic dysfunction–associated steatotic liver disease; PSC, primary sclerosing cholangitis.

Current evidence supporting the use of FMT in chronic liver disease is limited to small studies that suggest potential benefit across several indications, with the strongest data to date in cirrhosis and alcohol-associated liver disease (ALD) (Figure 2).

### Cirrhosis and hepatic encephalopathy

There are 4 published trials of FMT in patients with cirrhosis and prior overt hepatic encephalopathy (HE)—all 4 trials evaluated safety as the primary outcome (Table 1).^13^,^14^,^15^,^17^ Three of the trials were randomized and placebo-controlled. There were 10–60 patients enrolled in each trial, with a total of 75 patients receiving FMT across all 4 trials. In all the trials, there were mild adverse events attributed to FMT, largely gastrointestinal symptoms similar to the FMT literature for recurrent *Clostridioides difficile* infection (CDI). There was one serious adverse event attributed to FMT: transmission of extended-spectrum beta-lactamase-producing *Escherichia coli* bacteremia through FMT.^16^ Testing for this pathogen specifically has since been mandated for those conducting FMT research by the United States Food and Drug Administration (FDA).^26^ A multicenter retrospective observational study found that 5 serious adverse events occurred among 63 patients with cirrhosis who underwent FMT for CDI.^27^ These patients did not have a history of HE, and most were compensated. There were no infections or deaths. Approximately one-third of patients in this study had at least one non-serious adverse event possibly related to FMT, most often abdominal pain or diarrhea.

**TABLE 1:** Trials of fecal microbiota transplant in chronic liver disease

Trial	Patient population	Study design	Route and administration	Primary outcome	Result summary
Bajaj et al., 2017^13^	Men, cirrhosis, and recurrent HE, MELD <18, on lactulose and rifaximin; 20 patients	Randomized, placebo-controlled	Enema; single 90 mL dose; antibiotic pre-treatment	Safety (serious adverse events)	FMT enema was safe; significant improvement in the psychometric HE score with FMT
Bajaj et al., 2019^14^	Cirrhosis and recurrent HE, MELD score <18, on lactulose and rifaximin; 20 patients	Randomized, placebo-controlled	Capsules; single donor with high *Lachnospiraceae* and *Ruminococcaceae* abundance; single dose of 15 capsules	Safety (serious adverse events)	FMT capsules were safe; significant improvement in the Stroop test
Bloom et al., 2022^15^	Cirrhosis and prior overt HE, MELD score <18, on lactulose and rifaximin; 10 patients	Open-label, no placebo control	Capsules; 5 doses of 15 capsules (24 g each) on days 1, 2, 7, 14, and 21; 5 different donors used	Safety (serious adverse events) change in psychometric HE score	FMT capsules were safe, except for one FMT-related serious adverse event;^16^ significant improvements in psychometric HE score with FMT
Bajaj et al., 2025^17^	Cirrhosis and prior overt HE, on lactulose and rifaximin; 60 patients	Randomized, placebo-controlled	Capsules and enema; 2 donors with high *Lachnospiraceae* and *Ruminococcaceae* abundance; both routes deliver 2.5×10^12^ bacteria	Safety (adverse events)	FMT capsules and enema were safe; FMT groups were significantly less likely to experience overt HE than the placebo
Bajaj et al., 2021^18^	Alcohol use disorder and ALD cirrhosis, and active problem drinking; 20 patients	Randomized, placebo-controlled	Enema; single 90 mL dose	Safety (serious adverse events)	FMT enema was safe; FMT reduced alcohol cravings and urinary biomarkers of alcohol use
Ichim et al., 2025^19^	Men, ALD cirrhosis without ongoing alcohol use	Open-label, non-randomized, contemporary control arm	Fresh FMT via colonoscopy; single dose of minimum 70 g	Not specified	FMT group with improved grades of encephalopathy (did not report Stroop test scores)
Sharma et al., 2022^20^	Severe alcohol-associated hepatitis with ACLF	Open-label, non-randomized, contemporary control arm	Nasojejunal FMT; single dose	Survival at 28 and 90 d	Survival at 28 and 90 d was significantly better in the FMT arm
Groenewegen et al., 2025^21^	MASLD (degree of fibrosis not reported) as defined by ultrasound or FibroScan CAP >278 dB/m	Randomized, allogenic or autologous FMT; 20 patients	Upper endoscopy administration into the duodenum; 3 treatments of 60 g at baseline, week 3, and week 6	Change in liver fat per MRI-PDFF over 12 weeks	FMT did not affect liver fat, glucose tolerance, liver biochemistries, or microbiome composition
Craven et al., 2020^22^	Non-alcoholic fatty liver disease, per the AASLD definition, excluded diabetes requiring insulin	Randomized, allogenic vs. autologous FMT; 21 patients	Nasoduodenal FMT; 3 donors; single 2 g dose administered into the duodenum	Decrease in insulin resistance	No significant change in insulin resistance or hepatic steatosis
Leong et al., 2020^23^	Obese adolescents	Randomized, placebo-controlled; 87 patients	Capsules; 8 donors; single dose of 28 capsules (22 g) over 2 d from 4 same sex donors (7 from each donor)	Body mass index standard deviation score	No effect of FMT on body mass index standard deviation score
Chauhan et al., 2021^24^	HBeAg-positive patients, despite antiviral treatment for >1 y; 14 patients	Non-randomized, contemporary control arm	Nasoduodenal FMT; single donor; monthly 30 g dose of fresh stool into the duodenum for 6 doses	Loss of HBeAg and HBsAg	2/12 FMT patients had HBeAg clearance, and none had HBsAg clearance; none cleared in the contemporary control group
Allegretti et al., 2019^25^	Primary sclerosing cholangitis with inflammatory bowel disease, no cirrhosis; 10 patients	Open-label, no placebo control	FMT via colonoscopy; single donor; single 90 mL dose	Safety	FMT was safe, no related adverse events; 3/10 experienced a ≥50% decrease in alkaline phosphatase

Abbreviations: AASLD, American Association for the Study of Liver Diseases; ACLF, acute-on-chronic liver failure; ALD, alcohol-associated liver disease; CAP, controlled attenuation parameter; FMT, fecal microbiota transplantation; HBeAg, hepatitis B e antigen; HBsAg, hepatitis B surface antibody; HE, hepatic encephalopathy; MASLD, metabolic dysfunction–associated steatotic liver disease; MRI-PDFF, magnetic resonance imaging–proton density fat fraction.

While not powered to evaluate efficacy, all 4 pilot trials demonstrated a signal that cognitive function improves with FMT.^13^,^14^,^15^,^17^ However, there were conflicting results between trials in which cognitive test improved, either psychometric HE score alone, Stroop test alone, or both. It is currently unknown if these conflicting results are meaningful or the consequence of underpowered analyses. The psychometric HE score and Stroop tests evaluate slightly different cognitive domains. It has been suggested that the route of FMT administration (capsule vs. enema) may differentially influence certain cognitive areas over others; however, in the largest pilot trial, which employed both capsule and enema FMTs, there was no interaction between route and improvement in psychometric hepatic encephalopathy score (PHES) versus Stroop tests.^17^

Specific FMT-induced microbiome changes have been associated with improved cognition in patients with cirrhosis and HE. FMT enemas in patients with cirrhosis led to an increase in fecal *Ruminococcaceae* abundance, a genus known to produce SCFAs, which may, in turn, improve intestinal barrier function.^13^,^28^ However, this increase in *Ruminococcaceae* disappeared by 1 year post-FMT despite preserved cognitive improvements, so it is unclear if *Ruminococcaceae* were driving the cognitive improvement.^29^ In one study of FMT capsules, FMT was associated with an increase in duodenal *Ruminococcaceae*, an increase in antimicrobial proteins, an increase in tight junction protein E-cadherin expression, and a decrease in serum IL-6 and lipopolysaccharide (LPS)-binding protein.^7^,^14^ Altogether, these findings suggest the possibility of oral FMT improving duodenal barrier function and decreasing the translocation of bacteria or bacterial products. In another study of FMT capsules, cognitive test scores were associated with the abundance of 2 *Bifidobacterium* species, both known to produce SCFAs, and the FMT donor with the poorest cognitive outcomes in recipients had the lowest fecal SCFA levels.^15^ In the most recent THEMATIC trial of both FMT enemas and capsules, high post-FMT abundance of *Bifidobacteriaceae* appeared protective of subsequent overt HE episodes.^17^ Furthermore, patients who received FMT and had increased abundance of *Lachnospiraceae* and decreased abundance of *Lactobacilliaceae* were protected from subsequent HE.^17^ A recent conference abstract suggests that jejunal FMT in cirrhosis may reduce serum ammonia levels, which could have an impact on HE.^8^ However, the 4 published FMT trials discussed in detail in this section either do not mention ammonia levels or found no change. These conflicting results in ammonia testing may be due to the technical challenges with ammonia testing, the type of tissue being tested (stool vs. blood), or because FMT is not influencing ammonia metabolism.

From these trials, it appears that FMT-induced microbiome changes may be treating HE; however, the precise or singular mechanism of action remains elusive. Each trial noted different taxa that increased abundance with FMT and were associated with clinical efficacy. These conflicting results are possible for several reasons: first, these studies are small and likely underpowered to definitively evaluate microbiome changes with FMT; second, different FMT donors or lots were used in each trial, which impacts FMT composition and subsequent microbiome effects; third, it is possible that the functional niche matters more than the specific taxa, and many of the highlighted taxa play a role in SCFA metabolism.

### Alcohol-associated liver disease

Several recent studies have highlighted the strong association between the gut microbiome and ALD, mainly via the production of certain hepatotoxins (candidalysin, cytolysin), altered immune response, and increased intestinal permeability.^30^,^31^,^32^,^33^ In particular, a study of over 100 patients with alcohol use disorder found that duodenal mucosa-associated microbiota composition and increased intestinal translocation of microbes and microbial products were associated with progressive ALD.^30^

Given this association, microbiome manipulation via FMT has been considered and has been successful in preclinical models. Ferrere et al.^34^ found that some mice developed liver disease in response to alcohol, but another group of mice was resistant to alcohol liver disease, even with the same exposure. A principal coordinates analysis (PCoA) plot demonstrated that the fecal microbiome differed substantially (*p*<0.001) between alcohol-sensitive and alcohol-resistant mice, as did cecal bile acid composition. Several enteric markers also suggested increased intestinal permeability, specifically microbially mediated markers, in alcohol-sensitive mice. Furthermore, the authors found that FMT with stool from the alcohol-resistant mice was able to engraft in alcohol-sensitive mice, and this prevented the alcohol-sensitive mice from developing steatosis and hepatitis in response to alcohol. In other words, the protective microbiome was transferable. Liu et al.^35^ found that FMT or a compound probiotic alleviated acute alcohol-associated liver injury in mice, apparently by upregulating tight junction protein expression, decreasing gut translocation of bacterial products, modulating immune response, and decreasing hepatic lipid accumulation by regulating fatty acid metabolism-associated genes. Zhang et al.^36^ identified yet another possible mechanism by which FMT could impact ALD: FMT enriched to produce urolithin A was able to ameliorate ALD in mice, via attenuation of endothelial reticulum stress. Finally, FMT may ameliorate ALD beyond its direct impact on the liver, and rather through its impact on alcohol craving and consumption. Wolstenholme et al found that FMT substantially reduced alcohol intake (*p*=0.006) in mice, possibly the result of differentially expressed genes involved in immune response, inflammation, oxidative stress, and epithelial cell proliferation in the small intestine.^37^ Preclinical models have suggested potential efficacy of FMT in treating ALD, through several possible mechanisms.

FMT has also undergone pilot testing in human clinical trials of ALD. In a randomized trial of a single FMT enema, 10 patients with ALD received FMT and subsequently had reduced alcohol cravings, urinary biomarkers of alcohol use, and alcohol-associated adverse events.^18^ While the exact mechanism of action could not be determined from this pilot study, patients who received FMT had a change in fecal microbiota composition, reduction in serum IL-6 and lipopolysaccharide-binding protein, and increased plasma butyrate. FMT also led to clinical improvement in a small open-label pilot of severe alcohol-associated hepatitis.^20^ Finally, FMT via colonoscopy led to improved HE scores and lower systemic inflammation biomarkers in a small non-randomized open-label pilot in patients with ALD cirrhosis.^19^ A meta-analysis of FMT in severe alcohol-associated hepatitis evaluated 8 studies with 444 patients and found improved 30 and 90-day survival, though not at 6 or 12 months.^38^ They found insufficient microbiome and mechanism data to meta-analyze those results.^38^ Of note, all of these studies were performed in India and used freshly prepared nasojejunal FMT, which can be logistically challenging and limits the external validity of these findings. Sensitivity analysis revealed that only retrospective studies drove the short-term mortality benefit, calling into question potential publication and other biases influencing these results.

### Metabolic dysfunction–associated steatotic liver disease

There have been well-documented associations between fecal microbiota composition and metabolic dysfunction–associated steatotic liver disease (MASLD), including MASLD progression to cirrhosis.^39^,^40^ Microbially generated products lead to increased intestinal permeability and are associated with liver fibrosis progression in MASLD.^41^ These factors promoted interest in microbial manipulation to treat and prevent the progression of MASLD. In a high-fat diet mouse model of MASLD, FMT from healthy mice changed fecal microbiota composition, increased butyrate production and intestinal tight junction protein ZO-1, and lowered endotoxemia and steatohepatitis.^42^ Another set of mouse experiments demonstrates that without dietary changes, FMT does not improve the progression of MASLD. Mitsinikos et al.^43^ found that 2 different dietary interventions changed microbiome composition and metabolic function, as well as improved hepatic steatohepatitis. However, when these diet-altered microbiomes were transplanted into MASLD mice, who remained on a high-fat diet, those transplanted microbiomes did not improve hepatic steatohepatitis. These animal experiments suggest that FMT alone may not be sufficient to treat MASLD; rather, dietary interventions at a minimum may also be required, suggesting that microbiome treatments have an adjunct role in chronic multifactorial disorders.

The results from 3 pilot clinical trials suggest that FMT monotherapy is unlikely to be sufficient to treat MASLD. In a randomized controlled trial of 20 patients with MASLD, FMT did not affect hepatic steatosis, glucose tolerance, liver biochemistries, or microbiome composition.^21^ In 15 patients with MASLD who received FMT from a healthy donor, the FMT improved small intestinal permeability, but not insulin resistance or hepatic steatosis.^22^ FMT in adolescents showed no change in weight loss, liver injury, or metabolic parameters except for a reduction in abdominal adiposity.^23^ A placebo-controlled trial of FMT in obese individuals without MASLD also found no weight loss with FMT alone.^44^ These heterogeneous animal and human studies suggest that if there are microbiome changes with FMT in MASLD, they may simply be associated with FMT and not cause hepatic or systemic changes. Two meta-analyses report insufficient data on FMT to treat MASLD to evaluate its efficacy.^45^,^46^ However, one recent meta-analysis of a small number of studies found that FMT reduced liver biochemistries and proton density fat fraction, and another meta-analysis found reduced insulin resistance.^47^,^48^ These additional data are from a few small trials and would require further validation and improvement in important clinical endpoints to be promising.

### Other

FMT has been evaluated in a smattering of other liver diseases. A small pilot study suggested FMT may lead to hepatitis B virus (HBV) eAg clearance, though this only occurred in 2 of 12 patients.^24^ Given the influence of FMT on bile acid metabolism, FMT has also been trialed in cholestatic liver disease. In a 10-patient study, 3 patients with primary sclerosing cholangitis had a decrease in alkaline phosphatase levels.^25^ In a pig model of total parental nutrition liver injury, FMT reduced intestinal villous atrophy and cholestasis.^49^ Finally, in a mouse model, FMT appeared to ameliorate autoimmune hepatitis.^50^ These non-cirrhosis, MASLD, and ALD indications are quite early in their consideration.

### Future state of the art

Future studies of FMT in chronic liver disease should focus on the indications with the greatest unmet therapeutic needs, with high incidence and prevalence, as well as the indications with the most promising preliminary data for efficacy. These 2 indications are cirrhosis and ALD.

Fortunately, there are at least 2 actively enrolling large randomized trials of FMT in patients with cirrhosis.^51^,^52^ There is a multicenter Danish study called Cirrhosis and Fecal Microbiota Transplantation (ChiFT), which aims to enroll 220 patients with acutely decompensated cirrhosis to FMT versus placebo (NCT04932577). The primary outcome is time to new decompensation or death, and many of these patients will have overt HE as their decompensation or a history of HE. They will, in addition, evaluate subsequent overt HE and cognitive function in follow-up as secondary endpoints. The PROFIT study, or PROspective, randomized placebo-controlled feasibility trial of Fecal microbiota Transplantation in cirrhosis, is a United Kingdom study of 24 patients randomized to FMT versus placebo. There is an actively enrolling follow-up study, PROMISE, which has a much larger sample size of 300 patients with cirrhosis (NCT06461208).^53^ The primary outcome of PROMISE is time to first infection requiring hospitalization. PROMISE will, in addition, evaluate important secondary outcomes of hepatic decompensation and antimicrobial resistance (AMR) development.

These multicenter trials will give us higher-quality, sufficiently powered evidence about the efficacy and safety of FMT in cirrhosis. These studies will likely also give us insights into how FMT behaves in important subgroups—for example, by sex, age, cirrhosis etiology, decompensation type, interactions with specific medications (eg, lactulose, rifaximin), and baseline and follow-up microbiome compositions.

There are ongoing trials of FMT in patients with ALD. The IMPACT study, or Intestinal Microbiota Transplant in Alcohol-Associated Liver Disease, is a US randomized controlled trial of oral FMT capsules in 80 patients with advanced ALD and active ongoing alcohol use, with 3 primary outcomes—each evaluating alcohol use.^54^ There is also an Indian randomized trial of duodenal FMT delivered via endoscope in 54 patients with alcohol-associated cirrhosis and active drinking, with the primary outcome of abstinence at 4 weeks.^55^ Another US randomized controlled trial is evaluating oral FMT capsules in 50 patients with severe alcohol-associated hepatitis, with several primary outcomes including 1-year patient survival.^56^ These studies will specifically evaluate the separate and related impacts of FMT on alcohol cravings and consumption, as well as the direct impact of FMT on hepatic function and decompensation in ALD.

It would be pertinent to investigate and analyze which organisms engraft in recipients using validated methods and hypothesize how engraftment influences clinical outcomes.^57^,^58^,^59^,^60^ Clinical trials of FMT should be paired with a detailed evaluation of the mechanism, such as evaluation of stool metabolites, intestinal permeability, immune response, and metabolic changes. Clinical studies should also be paired with experimental models that are able to elucidate the precise effects of FMT and its components. However, the primary animal model used in HE experiments is a bile duct ligation model, which interferes with the evaluation of microbiome therapeutics.^61^

## VARIABILITY IN FMT COMPOSITION AND PROCESSING

**FIGURE 3:**
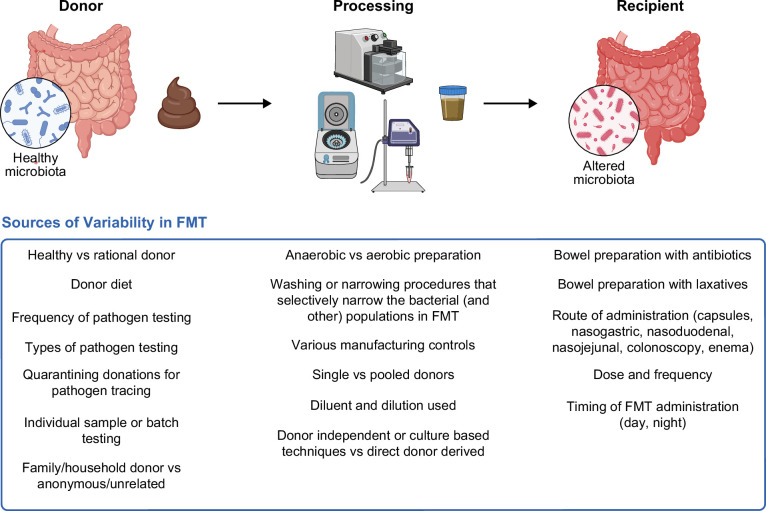
Sources of variability in FMT. Created in BioRender. Bloom, P. (2025), https://BioRender.com/m1veep0. Abbreviations: FMT, fecal microbiota transplantation.

Stool banks are facilities that screen potential FMT donors and process stool for the creation of FMT. There is no publicly available registry of stool banks in the United States, nor does the US Food and Drug Administration (FDA) maintain such a list.^62^ In one real-world study of 259 patients who received FMT for recurrent or refractory CDI (rCDI) from 2017 through 2019, 67% received FMT from a single US non-profit organization, OpenBiome, and 24% received FMT from other stool banks with “site-specific donor identification and screening techniques.”^63^ The majority of published FMT studies do not include key methodological details about FMT preparation.^64^ In the United States, providers are able to supply stool bank-generated FMT to their patients to treat rCDI without the regulatory burden of holding an investigational new drug (IND) application (discussed further in the Regulation section below).^65^ There is variability across US stool banks in FMT composition, processing, and risk mitigation strategies, without a clear purpose, which may lead to variability in product composition, efficacy, and safety (Figure 3).^62^,^66^ Without a publicly available registry of stool banks, it is challenging to quantify this heterogeneity, but some variable factors are known. First, some stool banks perform pathogen testing with every donation, while others perform pathogen testing on only a fraction of donated samples. Repeated testing increases cost, which limits feasibility. Second, there is variability around the practice of quarantining donations for the purpose of potential subsequent pathogen tracing. Third, comprehensive standardized donor testing is expensive and performed variably and not completely regulated.^66^ Most assays for pathogens are validated in active infection, not for asymptomatic carriers with possibly less fecal shedding.^67^ Pathogen testing is only for known pathogens, as unknown pathogens cannot yet be tested. Also, there is a delay in being able to test for novel pathogens [severe acute respiratory syndrome coronavirus 2 (SARS-CoV-2) and monkeypox are recent examples].

Despite this heterogeneity in production practices at stool banks, OpenBiome, for a decade, provided most of the FMT product used nationally, with over 72,000 treatments supplied to over 1300 clinical sites.^68^ As of 2025, there are 2 FDA-approved non-FMT microbiome therapies for rCDI. These are: fecal microbiota, live-jslm/Rebyota (approved November 2022), and fecal microbiota spores, live-brpk/Vowst (approved April 2023). In October 2024, OpenBiome closed its large FMT production and dispersal operations due to changes in the FDA regulation of FMT, suggesting the requirement of an IND and the 2 commercially available products (discussed further in the Regulation section below).

In a post-OpenBiome world, FMT in the United States is being generated largely by individual center-run stool banks. Some of the variability in FMT composition and processing from these stool banks only applies to FMT delivered for rCDI. When FMT is used for non-rCDI indications, the FDA requires an IND, which then demands a high bar of regulatory compliance, including a comprehensive list of donor screening measures. Given that all liver disease indications for FMT are experimental, any FMT used for liver disease has and will undergo the comprehensive donor recruitment and screening testing required by the FDA.

The future state of the art will move toward more homogeneous products, or intentional variability that serves some therapeutic or experimental purpose. Thankfully, there is now an international consensus protocol on how to screen and prepare FMT for clinical use.^69^ One Chinese program has a rigorous 5-step program (uniquely, they evaluate 16S for dysbiosis) with a 1.7% approval rate for donors, leading to high efficacy and low adverse events.^70^ There is also some evidence that anaerobic preparation of FMT increases obligate anaerobe viability, and many of those anaerobes serve important metabolic functions in the gut microbiome.^71^ The utility of anaerobically prepared FMT should be studied in liver disease.

Future state-of-the-art will also likely include alternative products, with increased homogeneity, that can presumably have an identical or similar impact on the recipient microbiome. Such alternative products include VE303, which has been trialed in cirrhosis and HE.^72^ VE303 is a defined consortium of 8 purified, clonal bacterial strains, originally isolated from human donors and now clonally produced from cell banks.^72^ VE303 was developed in a mouse model to treat CDI, and then in a phase II trial, was well tolerated and reduced the risk of recurrent *C. difficile* infection.^73^ Resistant starch is capable of yielding high SCFA production in healthy and other disease populations, and is being trialed now in cirrhosis.^74^ Another alternative is to transmit only bacterial spores and remove living bacteria with high ethanol concentrations, as is done by VOWST.^75^ VOWST is an oral microbiome therapy composed of Firmicutes bacterial spores, with other bacteria selectively removed via ethanol exposure, now FDA approved to prevent rCDI.^75^ Another option is REBYOTA, a microbiota suspension generated with good manufacturing practices from human donors.^76^ REBYOTA is administered rectally, each dose contains ≥10^7^ live organisms per milliliter(s) of suspension, and it is also FDA-approved to prevent rCDI.^77^ Finally, several other strategies for risk mitigation of FMT include spiking in infectious agents as checks, manufacturing controls, and washing.^78^

## VARIABILITY IN FMT ADMINISTRATION

The frequency, dose, and route of FMT administration are other ways in which FMT currently varies in liver disease studies without a clear purpose (Figure 3).

First, it is not clear how durable one-time FMT is in treating chronic liver diseases, and how many FMT doses will be needed and at what frequency. In chronic liver disease, presumably the disease-related factors influencing the gut microbiome persist after the administration of FMT, and may therefore lead the microbiome to return eventually to pre-FMT composition and function, suggesting the need for multiple doses. One study of 7 patients with cirrhosis who received a single FMT enema found similar fecal microbiome composition and sustained cognitive improvement at 1 year post-FMT, though this is a small sample with no control arm from which to draw conclusions.^29^ In the THEMATIC trial, patients received either 0, 1, 2, or 3 doses of FMT over 30 days, and there was no clear difference between 1, 2, and 3 doses in preventing HE recurrence at 6 months, though the study was not powered to answer the question of ideal dosing.^17^ A large study of FMT to treat CDI showed that FMT is typically effective for at least 1 year, even in a cohort with several CLD patients; though it is unclear if this finding applies to non-CDI diagnoses.^79^

Second, the ideal route of FMT in chronic liver disease is unknown. In a meta-analysis of 148 FMT studies for multiple indications, 30.4% were performed via colonoscopy, 25.6% were performed via mixed routes, 6.6% were performed via nasoduodenal tube, and 6.6% via nasogastric tube.^66^ Notably, oral FMT capsules have been found to be noninferior to colonoscopy in rCDI.^80^ This question was also investigated as a secondary endpoint in the THEMATIC trial, which suggested that enema and oral capsule administration are similarly effective, but the trial was not powered to answer this question.^17^

Third, the ideal FMT donor diet for chronic liver disease is unknown. FMT donor diet and, in turn, donor microbiome composition appear to matter, but are not given a spotlight in most pilot work. A vegan diet may be protective of steatosis (in certain conditions).^81^ The THEMATIC study used 2 donors, 1 vegan, and 1 omnivore, but was not powered to evaluate any potential difference in efficacy.^17^

The timing of FMT administration may matter, and has been largely ignored in FMT trials in liver disease to date. Time during the day of FMT, with reference to circadian rhythm, influenced the effect of FMT on MASH in the mouse model.^82^

The cost of FMT varies by stool bank, over time, number of administrations, and route of administration, but has recently been estimated at $3230 per single administration.^83^ The cost of this therapy, as well as the requirement for local expertise in indications, storage, and administration of FMT, will almost certainly have an impact on access moving forward.

Finally, the utility and safety of single versus pooled donors is unexplored in liver disease. One meta-analysis in ulcerative colitis found pooled donors to be superior to single donors in treatment response, but this has not become the norm in practice.^84^ Utilization of pooled donors adds complexity to pathogen tracing.

In the future state of the art, there should be intentional variability of FMT administration or standardized practice. An active trial of 100 patients in China aims to determine the ideal dose of FMT for cirrhosis and HE, comparing 400 and 800 mL of FMT via nasojejunal tube.^85^ There is not enough data available yet to convene a consensus conference, as has been done in inflammatory bowel disease, though this should be performed in the coming years when larger trials in liver disease have been completed.^86^

## IMPACT OF FMT ON ANTIBIOTIC RESISTANCE

Antibiotic resistance is a major and growing concern in patients with CLD, especially cirrhosis. A global epidemiological study of patients with cirrhosis found that 34% of bacterial infections were multidrug-resistant (MDR).^87^ It is possible that certain medications such as spontaneous bacterial peritonitis (SBP) prophylaxis commonly used in cirrhosis promote MDR development.^88^ Infection with an MDR organism, as opposed to antibiotic antibiotic-sensitive organism, portends a poorer prognosis.^87^,^89^

FMT has been investigated to reduce AMR, mostly outside the CLD population. In a small CDI trial and 2 larger hematological malignancy trials, FMT initially transferred some new anti-AMR genes from commensal organisms, then FMT led to a long-term (up to 9 mo) resistance to acquiring new MDR organisms typically associated with infection.^90^,^91^ In 2 pilot trials of non-liver disease populations, it seems that FMT may reduce antibiotic resistance when specific FMT strains outcompete existing MDR strains.^92^,^93^ Fecal microbiota spores, live-brpk (VOWST), has narrower composition than traditional FMT, also appears to reduce the abundance of anti-AMR genes.^94^ However, in one small pilot trial of FMT, there was no difference in extended-spectrum beta-lactamase (ESBL) *E. coli* carriage between FMT and placebo arms, though the study was not powered to detect a difference in that endpoint.^95^

Early data suggest that FMT may possibly improve antibiotic resistance in the gut microbiome of patients with CLD. An analysis of 2 pilot trials in cirrhosis has found an overall decrease in antibiotic resistance genes after FMT, though beta-lactamase and quinolone resistance genes actually rose, possibly due to pre-FMT antibiotic use.^96^

We need a clearer understanding of FMT’s impact on antibiotic resistance in liver disease, and strategies to optimize this for a reduction in future MDR organism infections. A review by Boolchandani et al.^97^ describes the many different ways antibiotic resistance can be determined (eg, culture vs. sequencing-based techniques), each method with different strengths and limitations. Likely, FMT trials in liver disease should employ at least sequencing-based techniques to identify AMR genes pre-FMT and post-FMT, with the caveat that this data does not perfectly capture the likelihood of future MDR organism infection.

## REGULATION OF FMT

It is important to clarify that the FDA regulates FMT differently for the indication of rCDI than it does for experimental conditions. Some of the FMT regulations that apply to rCDI do not apply to chronic liver disease. In 2014, the FDA exercised “enforcement discretion” with regard to using FMT to treat rCDI—the primary indication for FMT. This policy means that providers are allowed to provide FMT to patients with rCDI without holding an IND application. The provider is required to obtain informed consent from the patient, and the FMT donors must be screened and tested.^65^ In 2022, the FDA announced that enforcement discretion no longer applied to “centralized stool banks” like OpenBiome, leading to the end of their broad distribution of FMT in 2024.^62^ Smaller, hospital-based stool banks are still permitted to disperse FMT to patients with rCDI under the policy of enforcement discretion, without an IND.

For non-CDI conditions, such as any liver disease, the FDA requires an IND to use FMT. The FDA treats FMT as a drug, and therefore, the FDA has the authority to apply new drug regulatory requirements to FMT. If a sponsor is hoping to someday market FMT or a similar therapeutic for a chronic liver disease condition, they would need to apply for a biologics license application, which must demonstrate that the product is “safe, pure, and potent.”^62^ Due to the inherent complexity of biological products like FMT, the FDA cannot require that the product be identical each time. Instead, the FDA relies on stringent controls of the manufacturing process to ensure consistency across lots.^62^ Given the high degree of regulation and the relatively early stages of research into FMT for liver diseases, a long road lies ahead for approval of FMT for chronic liver disease.

Perhaps this regulatory journey will be different outside the United States. While the US FDA regulates FMT as a drug, there has historically been a wide range of designations for FMT in Europe, including tissue, unlicensed medicinal product, and others.^62^ The FDA has rejected treating FMT as a human tissue because its contents are not actually human in origin.^62^ In 2024, the European Union passed a regulation for substances of human origin, of which FMT is included. This regulation is aimed at ensuring the safety of the therapy and the more uniform management of FMT and other similar products across the European Union. While this regulation is still in its early days, there is optimism that this regulatory framework will not hinder research into non-rCDI conditions for FMT, such as liver disease.

There are no signs that the regulation of FMT for liver diseases will change in the United States. Future studies will require an IND and eventual biological license application for marketing approval. Maor et al.^98^ recently proposed a new system of regulation for living bacterial products, which assigns stringency of regulation based on risks of the product as well as the risk level of the patient population.

Since there are two FDA-approved microbiome products for rCDI, there is a potential for using these therapies off-label for chronic liver disease indications. While off-label use is technically allowed by the FDA, there are inherent ethical, financial, and efficacy implications for using these therapies for any liver disease. We would suggest that these therapies be used only under research settings under the purview of an IRB and possibly an IND if required by the FDA. This would enable the collection of necessary efficacy and safety data and advance the field further.

## CONCLUSIONS

There is optimism for FMT and other microbiome therapeutics in the treatment of several chronic liver diseases, especially cirrhosis, HE, and ALD. At the same time, there are many unanswered questions about how and in whom to use FMT in patients with chronic liver disease (Table 2). Larger studies at multiple sites are needed to better understand which indications and subpopulations hold promise for FMT. In particular, cirrhosis and HE, as well as ALD, appear to be promising indications for future powered trials. The mechanism of action of FMT in chronic liver disease remains incompletely understood, so future trials should include evaluation of the mechanism, including intestinal permeability, immune response, and metabolic changes. At present, there is some variability in the screening, processing, and administration of FMT. Some of this variability is inherent to the nature of FMT, but some of the variability could be standardized to optimize safety and efficacy. For example, future studies may consider a focus on oral encapsulated FMT, as this is likely to be most feasible moving forward. Ultimately, we may find that narrowed and donor-independent microbiome therapeutics are superior tools to provide a consistently effective result in CLD. In that scenario, FMT would become largely an investigative tool. Regulation of FMT for CLD indications in the United States will continue to require the rigid regulatory framework of other drugs, requiring an IND.

**TABLE 2:** Unanswered questions in fecal microbiota transplant for chronic liver disease

Category	Unanswered questions	Future studies
Evidence supporting the use of FMT in chronic liver disease	● Is FMT effective in preventing overt HE? ● Is FMT effective for improving cognitive function in patients with HE? ● Is FMT effective for preventing infections such as SBP in patients with cirrhosis? ● Is FMT effective for preventing decompensation in patients with cirrhosis? ● What are the mechanisms of action by which FMT improves outcomes in cirrhosis? (and could this mechanism be distilled into a narrowed/reproducible microbiome therapeutic?) ● Is FMT effective for decreasing alcohol cravings in patients with ALD? ● What are the mechanisms of action by which FMT impacts alcohol cravings?	Multisite trials are powered to evaluate the efficacy of FMT, paired with a detailed evaluation of the mechanism, including intestinal permeability, immune response, and metabolic changes.
Variability in FMT composition and processing	● Is there any utility to anaerobic preparation of FMT in chronic liver disease? ● Is there an alternative product that can be donor-independent and identical with each batch that could replace FMT for each liver disease indication?	It is unlikely that there will be a trial powered to compare anaerobically prepared versus aerobically prepared FMT in liver disease. Alternatives include using anaerobically prepared FMT moving forward as the only FMT arm in liver disease trials, as well as further mechanistic studies in preclinical models and cohort studies to attempt to understand the importance of anaerobes in this population. As for alternative products, as the mechanism of action of FMT is better understood in liver disease, attempts should be made to narrow microbiome therapeutics to target these mechanisms, and powered trials should be performed to evaluate the novel, reproducible therapies.
Variability in FMT administration	● What dose and frequency of FMT is needed to treat chronic liver diseases? ● What is the ideal route of administration of FMT to treat chronic liver diseases? ● Does the FMT donor diet impact efficacy in chronic liver disease? ● Does timing of FMT administration impact efficacy in chronic liver disease? ● Is there utility in pooled FMT from multiple donors to treat chronic liver disease?	Further evidence of a short-term benefit of FMT in liver disease is still needed. If short-term benefit is proven, future studies will need to increase the duration of follow-up to at least 1 year to understand the duration of clinical benefits and microbiome changes. The 2 large ongoing trials of FMT in cirrhosis are evaluating encapsulated FMT, which is also likely the most feasible route for future implementation.^44^,^45^ In the coming 5 years, there will be sufficient available evidence to convene a consensus conference to determine the ideal ways to administer FMT in liver disease.
Impact of FMT on antibiotic resistance	● Does FMT impact carriage of antimicrobial resistance genes in chronic liver disease? ● Does FMT impact the rate of future MDR infections in chronic liver disease?	FMT trials in liver disease should employ sequencing-based techniques to identify AMR genes pre- and post-FMT. The ongoing PROMISE trial is evaluating the effect of FMT on time to first infection requiring hospitalization, and will specify infections with MDR organisms.

Abbreviations: ALD, alcohol-associated liver disease; AMR, antimicrobial resistance; FMT, fecal microbiota transplantation; HE, hepatic encephalopathy; MDR, multidrug-resistant; SBP, spontaneous bacterial peritonitis.
